# Catecholamine-Induced Multiorgan Failure and Arterial Thrombosis: A Rare Manifestation of Pheochromocytoma in the Setting of Community-Acquired Pneumonia

**DOI:** 10.7759/cureus.104936

**Published:** 2026-03-09

**Authors:** Ahd Elmahi, Maitha Humaid Ibrahim, Rehab Mohd Jamali, Tahsina Tajnin Sadia, Mohamed Ragab Radwan

**Affiliations:** 1 College of Medicine, University of Sharjah, Sharjah, ARE; 2 Department of Emergency Medicine, Al Qassimi Hospital, Sharjah, ARE

**Keywords:** arterial thrombosis, cardiomyopathy, multi-organ failure, pheochromocytoma, septic shock

## Abstract

Pheochromocytoma is a rare catecholamine-secreting tumor that has a highly variable presentation and may result in life-threatening complications. In the most severe cases, massive release of catecholamines can lead to refractory shock, acute cardiomyopathy, and multiorgan failure. The diverse clinical manifestations of pheochromocytoma often make diagnosis challenging in critical care settings. We report a 25-year-old woman who presented with acute abdominal pain, vomiting, pleuritic chest pain, and a productive cough and was initially managed as community-acquired pneumonia complicated by septic shock. She rapidly deteriorated, requiring intubation, vasopressor support, and advanced cardiac life support following cardiac arrest. Bedside echocardiography showed severe global left ventricular systolic dysfunction with an estimated ejection fraction of 20%-25%. During her stay in the intensive care unit, a femoral catheterization was performed for vascular access and hemodynamic monitoring. However, in the context of a catecholamine excess state, she subsequently developed acute ischemia of the left lower limb. Computed tomography angiography (CTA) showed extensive arterial occlusion, and an incidental 4 × 4 cm left adrenal mass was discovered. Follow-up biochemical testing showed markedly elevated plasma metanephrines and normetanephrines, supporting the diagnosis of pheochromocytoma. Despite treatment with thrombectomy, her limb ischemia was irreversible, necessitating an above-knee amputation. With supportive care and initiation of α-adrenergic and β-adrenergic blockade, her cardiac and renal function gradually improved. She was stabilized and referred for definitive surgical management. This case highlights that pheochromocytoma crisis may mimic septic shock and can present with severe cardiomyopathy and limb-threatening arterial thrombosis. Early consideration of catecholamine excess in unexplained refractory shock or multiorgan failure is important, since early diagnosis and targeted treatment can be lifesaving and help prevent irreversible complications.

## Introduction

Pheochromocytoma is a rare but potentially life-threatening catecholamine-secreting tumor that arises from the chromaffin cells of the adrenal medulla. While its prevalence remains low, with an estimated 19.8 cases per one million individuals [1], it carries significant clinical importance due to the systemic effects of excess catecholamines. Sustained or episodic release of catecholamines can lead to marked cardiovascular and metabolic disturbances, including severe hypertension, arrhythmias, myocardial dysfunction, arterial thrombosis, and end-organ ischemia. The classic clinical presentation consists of episodic headache, palpitations, diaphoresis, and paroxysmal hypertension. However, many patients present with nonspecific or atypical features, which may delay recognition. In severe cases, physiological stressors such as infection, surgery, or trauma can precipitate a sudden surge in catecholamine release, resulting in a life-threatening condition known as pheochromocytoma crisis [2]. This presentation may manifest with circulatory collapse, cardiogenic shock, or multiorgan dysfunction, closely mimicking septic shock, myocarditis, or other critical illnesses encountered in emergency and intensive care settings [3].

In acute settings, biochemical confirmation and imaging are often conducted only after initial stabilization; hence, most cases are incidental findings during evaluation for unexplained multiorgan dysfunction. Early recognition is essential, as appropriate α-adrenergic blockade followed by definitive surgical resection significantly improves clinical outcomes. We report the case of a 25-year-old female who initially presented with community-acquired pneumonia and septic shock, with subsequent rapid progression to cardiogenic shock and acute arterial thrombosis. Further evaluation ultimately revealed an underlying pheochromocytoma. This case underscores the importance of considering catecholamine excess in patients with unexplained circulatory collapse or atypical shock states.

## Case presentation

A 25-year-old female presented to the emergency department with a one-day history of sudden diffuse abdominal pain, multiple episodes of vomiting (>10), pleuritic chest pain, and a new productive cough. She denied fever, diarrhea, urinary symptoms, weight loss, or sick contacts. She has a known history of hypothyroidism but is poorly compliant with her medication (levothyroxine 25 µg daily). There was no history of cardiovascular disease, diabetes, thromboembolic events, or allergies.

On presentation, the patient was mildly hypoxemic (peripheral capillary oxygen saturation (SpO_2_) 90% on room air), tachycardic (heart rate (HR), 124 beats/min), and tachypneic (respiratory rate (RR), 30 breaths/min). Blood pressure was normal. On physical examination, she had reduced air entry with crackles on the left side and generalized abdominal tenderness without peritoneal signs. Extended Focused Assessment with Sonography for Trauma (eFAST) showed minimal fluid collection in the pelvic region. Neurological examination was unremarkable. Initial laboratory investigations indicated severe multisystem dysfunction, including marked leukocytosis, metabolic acidosis with hyperlactatemia, acute kidney injury, transaminitis, and marked myocardial injury (Table 1). Chest radiography showed left-sided consolidation with bilateral patchy opacities. Eventually, the patient became hypotensive. Fluid resuscitation and empiric broad-spectrum antibiotics were initiated for presumed community-acquired pneumonia complicated with septic shock. Blood cultures subsequently confirmed the diagnosis of community-acquired methicillin-resistant *Staphylococcus aureus* (MRSA) pneumonia.

**Table 1:** Selected laboratory investigations on admission.

Test	Result	Reference Range
White blood cell count (×10³/µL)	25.4	4.0-10.0
Arterial lactate (mmol/L)	9.8	<2.0
Creatinine (µmol/L)	155	53-97
Aspartate aminotransferase (IU/L)	788	15-37
Alanine aminotransferase (IU/L)	677	14-59
Troponin-I (ng/L)	1,891	<60
Creatine kinase (IU/L)	>1,000	26-192
C-reactive protein (mg/L)	51.8	<3.0
Procalcitonin (ng/mL)	2.11	<0.10
Thyroid-stimulating hormone (mIU/L)	33.3	0.4-4.0
Free T4 (pmol/L)	5.9	7.0-19.0

Despite aggressive resuscitation, the patient rapidly deteriorated (marked tachycardia; HR, 180 beats/min), requiring endotracheal intubation and vasopressor support. She subsequently sustained a cardiac arrest, with return of spontaneous circulation after six cycles of advanced cardiac life support. Post-resuscitation transthoracic echocardiography demonstrated severe global left ventricular systolic dysfunction with an estimated ejection fraction of 20%-25% and a collapsed inferior vena cava, consistent with stress-induced cardiomyopathy or fulminant myocarditis. The patient was transferred to the intensive care unit, sedated, and mechanically ventilated on high inotropic support.

**Figure 1:**
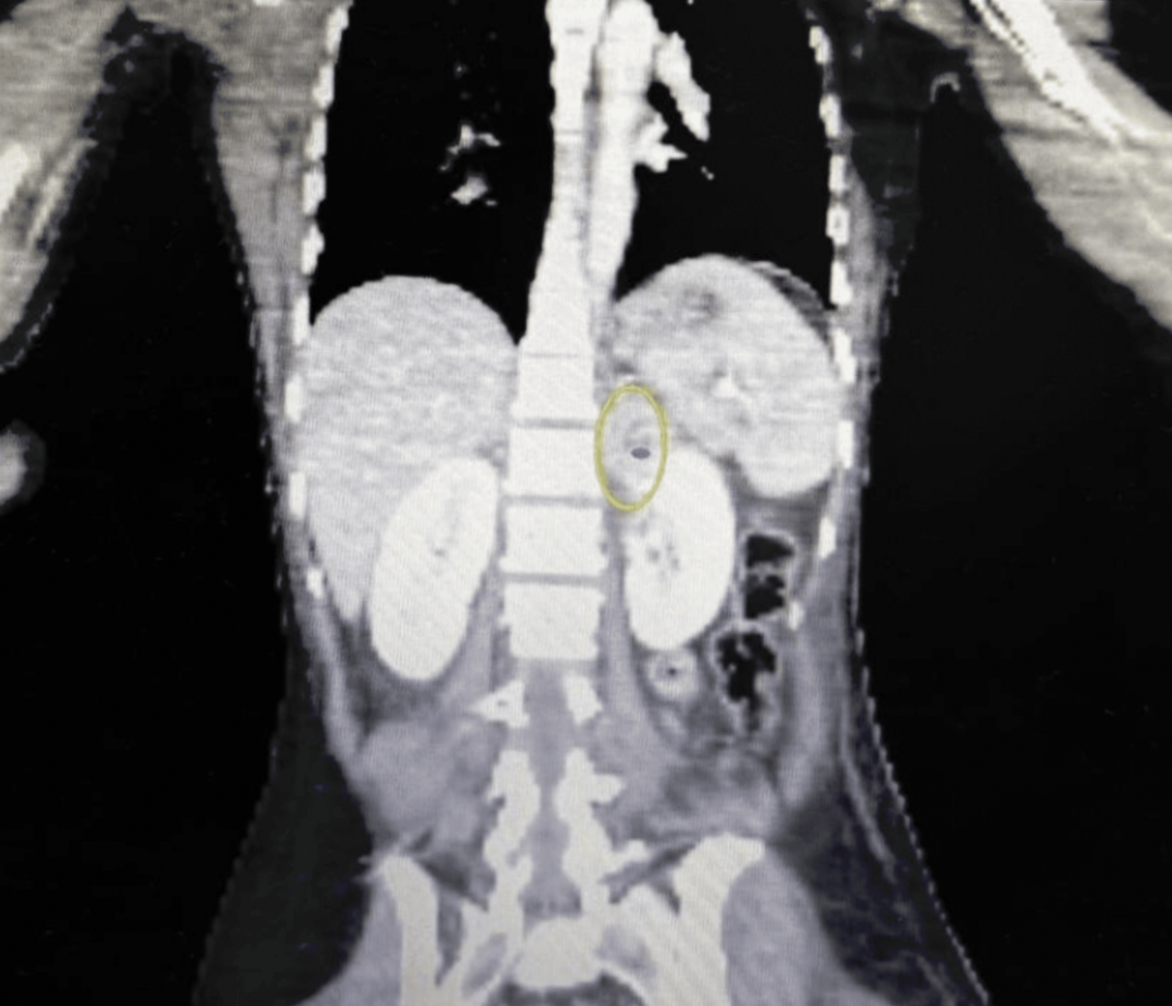
CT angiography showing a 4 × 4 cm left adrenal mass with peripheral arterial phase enhancement (yellow oval) and a central heterogeneous cystic component.

During her stay in the intensive care unit, the patient developed acute left lower limb ischemia following femoral arterial catheterization. Computed tomography angiography showed complete occlusion of the left external iliac, common femoral, superficial femoral, and profunda femoris arteries. Incidentally, a 4 × 4 cm left adrenal mass demonstrating avid peripheral arterial phase enhancement with a central heterogeneous cystic component was identified, raising strong suspicion for pheochromocytoma (Figure 1). Given the combination of refractory shock, severe cardiomyopathy, extreme tachycardia, metabolic acidosis, and arterial thrombosis, a catecholamine-mediated crisis was suspected.

Biochemical evaluation confirmed markedly elevated plasma normetanephrine, metanephrine, and 3-methoxytyramine, supporting the diagnosis of a catecholamine-secreting adrenal tumor (Table 2). A comprehensive thrombophilia and autoimmune workup was negative. The patient underwent limited aspiration thrombectomy; however, progressive irreversible ischemia ensued, necessitating a left above-knee amputation. The ischemic event was attributed to catecholamine-mediated vasoconstriction and thrombosis in the setting of a pheochromocytoma crisis.

**Table 2:** Plasma catecholamine and metanephrine profile.

Test	Result	Reference Range
Plasma normetanephrine (pg/mL)	1,798	<120
Plasma metanephrine (pg/mL)	145	<70
3-methoxytyramine (pg/mL)	25	<18

Following stabilization, her hemodynamic status improved, vasopressor support was discontinued, and cardiac, renal, and inflammatory parameters showed gradual recovery. She was initiated on α- and β-adrenergic blockade as tolerated, and her levothyroxine dose was optimized. After multidisciplinary review, she was deemed clinically stable for discharge. In view of suspected pheochromocytoma, the patient was advised to transfer to a specialized center for possible laparoscopic adrenalectomy. She elected to pursue further management in her home country. Endocrinology follow-up was arranged within one week to review metanephrine results and optimize α-blockade, and internal medicine follow-up was arranged at one month. The patient was discharged on prescribed medications and counseled regarding strict adherence and the need for timely treatment. She was advised to seek urgent medical attention should concerning symptoms recur.

## Discussion

Pheochromocytoma is a rare neuroendocrine tumor characterized by excessive catecholamine secretion by the chromaffin cells of the adrenal medulla. The classic triad of symptoms includes headaches, sweating, and heart palpitations, along with paroxysmal hypertension. However, in severe cases, significant episodic or sustained catecholamine release can lead to a pheochromocytoma multisystem crisis (PMC), resulting in hemodynamic instability, including hypotension, tachyarrhythmias, fever, encephalopathy, and multi-organ dysfunction. It may even progress to cardiogenic shock, circulatory collapse, and organ failure [2,4].

Our patient is a 25-year-old female with known hypothyroidism who initially presented with features suggestive of community-acquired pneumonia complicated by septic shock. Given her profound hyperlactatemia (9.8 mmol/L) and persistent hypotension, she was managed according to the Surviving Sepsis Campaign 2021 guidelines with intravenous fluids, high-flow oxygen, and early broad-spectrum antibiotics [5]. Despite appropriate interventions, her condition rapidly deteriorated, resulting in cardiac arrest and severe left ventricular dysfunction. The preceding infection and septic shock likely acted as physiological stressors, precipitating a pheochromocytoma crisis. The clinical course of this patient highlights the diverse manifestations of catecholamine excess and the diagnostic challenges encountered in critical care settings [6].

In pheochromocytoma, excessive catecholamine release disrupts cardiovascular function by overstimulating adrenoceptors in the heart and blood vessels. High catecholamine levels increase heart rate and contractility, leading to vasoconstriction of systemic and coronary blood vessels. This significantly raises myocardial oxygen demand and puts the heart at risk of ischemia and hypoxia. These mechanisms underlie catecholamine-induced cardiomyopathy and present as severe global left ventricular dysfunction, tachyarrhythmia, and cardiogenic shock, consistent with the acute cardiac collapse seen in our patient [7]. Another diagnostic consideration was the patient’s underlying thyroid disease. The presence of tachycardia, vomiting, and hemodynamic instability initially raised suspicion of thyroid dysfunction or effects of thyroxine therapy. However, laboratory results showed markedly elevated thyroid-stimulating hormone (TSH) with low free T4, consistent with hypothyroidism, which typically reduces sympathetic activity. This made thyroid storm unlikely and prompted consideration of other causes of sympathetic overactivity.

The patient’s clinical course was further complicated by acute left lower limb ischemia due to occlusion of the external iliac and femoral arteries. Elevated circulating catecholamines can induce intense α-adrenergic-mediated vasoconstriction and endothelial dysfunction, which have been associated with a prothrombotic tendency [8,9]. However, in this case, the development of arterial thrombosis was likely multifactorial. Profound shock may have contributed to low-flow states and endothelial injury, while recent femoral arterial instrumentation likely played a significant role in precipitating local thrombosis. Although limited proximal revascularization was initially attempted, rapid progression to fixed mottling and absent distal perfusion was consistent with Rutherford class III acute limb ischemia, necessitating an above-knee amputation as a life-saving measure [10].

The incidental discovery of a left adrenal mass on CT was a key diagnostic turning point, raising suspicion for pheochromocytoma. According to Endocrine Society guidelines, plasma-free or urinary fractionated metanephrines are recommended as the initial diagnostic tests due to their high sensitivity, followed by cross-sectional imaging for tumor localization [11]. On CT, pheochromocytoma typically appears as a well-defined adrenal mass with intense contrast enhancement and may demonstrate cystic or hemorrhagic features [12]. Definitive management involves surgical resection following appropriate preoperative preparation with α-adrenergic blockade for seven to 14 days, with β-blockers added after adequate α-blockade to control tachycardia. Volume expansion and careful perioperative monitoring are essential, and lifelong biochemical surveillance with plasma or urinary metanephrines is recommended to detect recurrence [11]. In patients with refractory cardiogenic shock, veno-arterial extracorporeal membrane oxygenation (VA-ECMO) may provide temporary circulatory support while allowing myocardial recovery [13]. In our case, ECMO was considered but not initiated due to resource limitations and partial stabilization after resuscitation.

Our patient’s presentation aligns with several documented cases of catastrophic pheochromocytoma. Rojbi et al. report the case of a 40-year-old woman who presented with new-onset heart failure (ejection fraction (EF) <20% with elevated biomarkers) initially thought to be due to viral myocarditis [14]. She was later discovered to have an adrenal pheochromocytoma, and after tumor resection, her cardiac function improved [14]. Another case of a 46-year-old female, reported by Chung et al., presented with refractory shock from cardiogenic shock with severely reduced left ventricular (LV) systolic function, metabolic acidosis, and markedly elevated troponin levels [15]. She was treated with mechanical circulatory support (VA-ECMO and Impella) until her condition stabilized. Unfortunately, the patient suffered from right lower limb gangrene due to the arterial limb of right femoral ECMO, eventually requiring above-knee amputation [15]. Vascular complications have also been reported in a 16-year-old female with pheochromocytoma who developed cardiogenic shock complicated by arterial thrombosis of the left lower limb following ECMO, necessitating above-knee amputation [16]. These cases mirror our patient's progression, demonstrating that extreme catecholamine excess can lead to profound cardiomyopathy, refractory cardiovascular shock, and vascular complications. 

## Conclusions

This case underscores the catastrophic potential of pheochromocytoma crisis, particularly when complicated by multi-organ failure, hemodynamic instability, and rare thrombotic complications like acute limb ischemia. The overlap of clinical features with septic shock complicates early diagnosis and often leads to delays. Excess catecholamine release not only causes severe cardiovascular compromise but can also promote a prothrombotic state, leading to vascular incidents. However, in this patient, the development of acute limb ischemia was likely multifactorial, with contributing factors including severe shock-related low-flow states and recent femoral arterial instrumentation in addition to possible catecholamine-mediated vascular effects. Prompt recognition, aggressive hemodynamic stabilization with the appropriate α-blockers, good supportive care, and timely surgical removal are critical for survival. This case emphasizes the need to consider pheochromocytoma crisis in patients presenting with unexplained shock, rapidly progressive organ failure, and atypical thrombotic manifestations. Early detection and management can greatly improve the overall outcome in this otherwise life-threatening yet treatable condition.

## References

[1] Vitturi G, Crisafulli S, Alessi Y (2025). Global epidemiology of pheochromocytoma: a systematic review and meta-analysis of observational studies. J Endocrinol Invest.

[2] Giraud R, Glauser A, Looyens C (2025). Pheochromocytoma multisystem crisis requiring temporary mechanical circulatory support: a narrative review. J Clin Med.

[3] Scholten A, Cisco RM, Vriens MR (2013). Pheochromocytoma crisis is not a surgical emergency. J Clin Endocrinol Metab.

[4] Newell KA, Prinz RA, Pickleman J, Braithwaite S, Brooks M, Karson TH, Glisson S (1988). Pheochromocytoma multisystem crisis: a surgical emergency. Arch Surg.

[5] Evans L, Rhodes A, Alhazzani W (2021). Surviving Sepsis Campaign: International Guidelines for Management of Sepsis and Septic Shock 2021. Crit Care Med.

[6] Tanriver Y, Betz MJ, Nibbe L, Pfluger T, Beuschlein F, Strowski MZ (2010). Sepsis and cardiomyopathy as rare clinical manifestations of pheochromocytoma: two case report studies. Exp Clin Endocrinol Diabetes.

[7] Szatko A, Glinicki P, Gietka-Czernel M (2023). Pheochromocytoma/paraganglioma-associated cardiomyopathy. Front Endocrinol.

[8] Duka I, Gavras I, Johns C, Handy DE, Gavras H (2000). Role of the postsynaptic α2-adrenergic receptor subtypes in catecholamine-induced vasoconstriction. General Pharmacology: The Vascular System.

[9] López García de Lomana A, Vilhjálmsson AI, McGarrity S (2022). Metabolic response in endothelial cells to catecholamine stimulation associated with increased vascular permeability. Int J Mol Sci.

[10] Björck M, Earnshaw JJ, Acosta S (2020). Editor's Choice: European Society for Vascular Surgery (ESVS) 2020 Clinical Practice Guidelines on the Management of Acute Limb Ischaemia. Eur J Vasc Endovasc Surg.

[11] Lenders JW, Duh QY, Eisenhofer G (2014). Pheochromocytoma and paraganglioma: an endocrine society clinical practice guideline. J Clin Endocrinol Metab.

[12] Corral de la Calle MA, Encinas de la Iglesia J, Fernández-Pérez GC, Repollés Cobaleda M, Fraino A (2022). Adrenal pheochromocytoma: keys to radiologic diagnosis. (Article in Spanish). Radiologia (Engl Ed).

[13] Rao P, Khalpey Z, Smith R, Burkhoff D, Kociol RD (2018). Venoarterial extracorporeal membrane oxygenation for cardiogenic shock and cardiac arrest. Circ Heart Fail.

[14] Rojbi I, Adel M, Affes M, Hantous S, Jrad M, Ben Nacef I, Khiari K (2021). Pheochromocytoma presenting as fulminant myocarditis mimicking COVID-19 pneumonia. Clin Case Rep.

[15] Chung PM, Yung KS, So D, Wong SK, Cheng LF (2025). Large pheochromocytoma presenting as refractory cardiogenic shock and multiorgan failure: a case report. Interv Radiol (Higashimatsuyama).

[16] Salameh M, Swizer K, Patel AP, Petrescu M (2025). Acute cardiogenic shock requiring extracorporeal membrane oxygenation secondary to catecholamine-secreting paraganglioma: a case report. Cureus.

